# The Role of Anti-Viral Effector Molecules in Mollusc Hemolymph

**DOI:** 10.3390/biom12030345

**Published:** 2022-02-23

**Authors:** Angus Watson, Jacinta Agius, Danielle Ackerly, Travis Beddoe, Karla Helbig

**Affiliations:** 1 Department of Physiology, Anatomy, and Microbiology, La Trobe University, Melbourne, VIC 3086, Australia; 18091537@students.latrobe.edu.au (A.W.); 19318704@students.latrobe.edu.au (J.A.); 2 Department of Animal, Plant and Soil Science, La Trobe University, Melbourne, VIC 3086, Australia; 19669349@students.latrobe.edu.au

**Keywords:** mollusc, abalone, hemolymph, galectin, lectin, antimicrobial peptide, antiviral defensin, virus

## Abstract

Molluscs are major contributors to the international and Australian aquaculture industries, however, their immune systems remain poorly understood due to limited access to draft genomes and evidence of divergences from model organisms. As invertebrates, molluscs lack adaptive immune systems or ‘memory’, and rely solely on innate immunity for antimicrobial defence. Hemolymph, the circulatory fluid of invertebrates, contains hemocytes which secrete effector molecules with immune regulatory functions. Interactions between mollusc effector molecules and bacterial and fungal pathogens have been well documented, however, there is limited knowledge of their roles against viruses, which cause high mortality and significant production losses in these species. Of the major effector molecules, only the direct acting protein dicer-2 and the antimicrobial peptides (AMPs) hemocyanin and myticin-C have shown antiviral activity. A better understanding of these effector molecules may allow for the manipulation of mollusc proteomes to enhance antiviral and overall antimicrobial defence to prevent future outbreaks and minimize economic outbreaks. Moreover, effector molecule research may yield the description and production of novel antimicrobial treatments for a broad host range of animal species.

## 1. Introduction

Immune defence systems differ between vertebrates and invertebrates; with vertebrates possessing both adaptive and innate immune systems, in comparison to invertebrates which possess only innate immunity [1]. The invertebrate circulatory fluid, termed hemolymph, is analogous to blood in vertebrates, and has also been shown to be vital to antimicrobial defence and nutrient distribution, containing immune cells named hemocytes, which secrete regulatory molecules termed effector molecules [2,3]. Thus, effector molecules in the proteome of invertebrates, such as proteins, antimicrobial peptides (AMPs), and lectins are likely to be key components of invertebrate antimicrobial defence [3].

The immune systems of invertebrates remain poorly understood despite the genomes of several model invertebrates having been sequenced, including the Common Fruit Fly (*Drosophila melanogaster*) and the nematode (*Caenorhabditis elegans*) [4,5]. Recent literature has described invertebrate antibacterial and antifungal defence, however, antiviral innate immunity studies are limited. Despite over twenty mollusc genomes being sequenced there is very little known regarding invertebrate antiviral responses in marine Mollusca phylum, such as the Pacific Oyster (*Crassostrea gigas*), which has been the focus of many genomic investigations, and the Blue Mussel (*Mytilus galloprovincialis*) from which many AMPs have been isolated and named [6,7,8]. 

Members of the Mollusca phylum represent great economic importance for both the international and the Australian aquaculture industry, though production is threatened by pathogens, such as viruses [9,10,11]. The most commonly farmed and wild-caught members of the Mollusca phylum include the bivalves such as oyster, clams, scallops, mussels, and the marine gastropod abalone [9,10,11,12], with abalone currently a significant contributor to aquaculture production in Australia [9]. The most abundantly reared abalone in Australia are the Victorian Green Lip Abalone (*Haliotis laevigata*), Black Lip Abalone (*Haliotis rubra*), and their hybrid, the Jade Tiger Abalone [10]. In 2022, the rearing of abalone is expected to contribute $140M to Australian aquaculture production, with an estimated growth of 38% to occur by 2025 [11]. Moreover, Tasmania leads the world in wild-caught abalone production, contributing to Australia’s aquaculture exports, which has a projected value of $1.35B AUD in 2025–2026 [11]. Pathogens pose extreme risks to abalone and other molluscs, offsetting aquaculture production as a result, which accounts for one third of global food production [9,10,11]. It is estimated that $2B US of aquaculture production is lost per year, with viruses being a major contributor to this figure [9,10,11,13]. Specifically, 10% of reared aquaculture species are lost due to pathogens each year, 90% of which pertain to viruses or bacteria [14]. Herpesviruses, a family of DNA viruses possessing an icosahedral structure, have been linked with recent outbreaks of disease in molluscs, with Haliotid Herpesvirus (HaHV1) infecting populations of Australian abalone [15]. Furthermore, HaHV-1 causes the neurological condition Abalone Viral Ganglioneuritis (AVG), and in the past has exhibited an approximate 90% mortality rate in Victorian species of abalone [16,17,18]. Moreover, Ostreid Herpesvirus (OsHV-1) has been associated with mass mortality in the molluscan class of Bivalvia, such as in *C. gigas* and the Blood Ark Clam *Scapharca broughtonii*, with studies showing up to 83% reduction in *C. gigas* production across five years in some areas [15,19,20]. 

Due to the limited research into mollusc innate pathways, viruses continue to affect host mortality and aquaculture production. Further research that identifies the effector molecules involved in mollusc antiviral defence and that describes their mechanism of action may benefit the industry by providing novel options for antiviral therapeutics. This review aims to introduce the current understanding of innate immunity in molluscs, and to describe the roles of known key effector molecules and their involvement or potential involvement in antiviral defence.

## 2. Interferon and Innate Immunity Signaling Pathways

Vertebrate immunity consists of two responses, the adaptive and the innate system. The innate response is activated quickly following infection, and ultimately leads to the production of the protein interferon (IFN), a cytokine and effector molecule involved in antiviral and inflammatory pathways [1]. This innate pathway is activated by the detection of pathogen-associated molecular patterns (PAMPs) such as aberrant viral nucleic acid from pathogens, which is recognised by pathogen recognition receptors (PRRs), such as Toll-Like Receptors (TLRs), Rig-Like Receptors (RLRs) and double stranded DNA sensors.

The recognition of PAMPs by PRRs triggers a signaling cascade which upregulates cytokines, including IFN [19,20]. Cytokines are secreted from infected cells and bind to other nucleated cells, instigating an antiviral state via activation of the Janus Kinase/Signal Transducer and Activator of Transcription (JAK-STAT) pathway, and promoting the upregulation of interferon-stimulated genes (ISGs) [21]. Subsequently, ISGs can act in an antiviral manner by disrupting various stages of viral life cycles, and providing a local antiviral environment [19]. Following viral challenge, the adaptive immune system of vertebrates can provide antiviral ‘memory,’ incorporating T cells, B cells, and antibodies, to support a quickened and strengthened immune response for future viral challenges [1]. 

On the contrary, invertebrate innate immunity is thought to be non-specific, is seemingly absent of IFN and adaptive immune mediated ‘memory’, and utilises limited ISGs that are homologous with those known to be present in vertebrates [22]. Given the limited research in this area, and the lack of experimental model systems, it is possible that soluble molecules similar to IFN and the downstream ISGs may exist, however, they remain elusive to date. It is therefore likely that other soluble molecules are vital for antiviral defence within invertebrate hemolymph, which is known to contain hemocytes to clear microbial infections via phagocytosis, as well as the secretion of effector molecules, such as antimicrobial peptides (AMPs) [23]. Additional effector molecules in the proteome of these organisms include proteins that are known to activate innate immune pathways (Dicer-2, Vago), as well as proteins which regulate immunological reactions and behave as additional PRRs (lectins) [3]. Recent literature has shown that mollusc innate systems diverge from model organisms and are more complicated [24,25]. As ocean environments can contain up to 1 million bacteria and 10 million viruses per millilitre, it is inferred that molluscs possess resilient and developed innate immune systems [26]. For example, the mollusc *C. gigas* genome encodes for more than 80 TLRs, whereas the insect *D. melanogaster* genome encodes for only 9 TLRs; however, the functionality of most of these TLRs is still unknown [27,28].

A significant component of invertebrate innate immunity is RNA interference (RNAi), a primitive system that promotes the degradation of viral mRNA [29]. The pathway involves the protein Dicer-2 cleaving viral dsRNA into fragments, that act as a template from which small interfering RNAs (siRNAs) (otherwise termed micro RNAs (miRNAs)) are transcribed [29,30]. Furthermore, siRNAs interact with the preRNA-inducing complex (RISC), which seeks complementary viral mRNA sequences and enables binding, thus inhibiting the mRNA and viral lifecycle [30]. Additionally, Dicer-2 upregulates the peptide Vago, via a pathway involving TRAF and REL2 [31]. Vago can induce the JAK-STAT pathway and upregulate ISGs such as vir1 and CG9080, which share no similarities to vertebrate ISGs [24,32,33]. Limited literature exists on mollusc antiviral transcriptional responses, or the related effector molecules, though a system has been described that is independent of RNAi, but does exploit Dicer-2 to detect replicating viruses [31]. This system requires further investigation, but implies the presence of novel antiviral defences that remain poorly understood in the molluscs. 

Transcriptome sequencing of *C. gigas* infected with Ostreid Herpesvirus (OsHV-1) suggests that the mollusc innate immune system contains homologous components of an ancient IFN pathway in hemocytes [7]. These components are vital in vertebrate antiviral defence and include PRRs such as TLRs and Rig-Like Receptors (RLRs), Stimulator of Interferon Genes (STING), IFN regulatory factors (IRFs), as well as ISGs such as viperin (RSAD2), a well-documented and highly conserved protein, which inhibits the replication of DNA and RNA viruses that infect humans [32,34,35]. Other OsHV-1 studies support the presence of a novel and secretable protein in hemolymph; specifically, a heat-stable protease susceptible factor that upregulates ISG transcription [32,33,35]. Further investigations into this factor may provide insights into the compound’s mode of action, thus allowing researchers to manipulate regulatory levels and may possibly lead to heightened immunity. 

**Figure 1:**
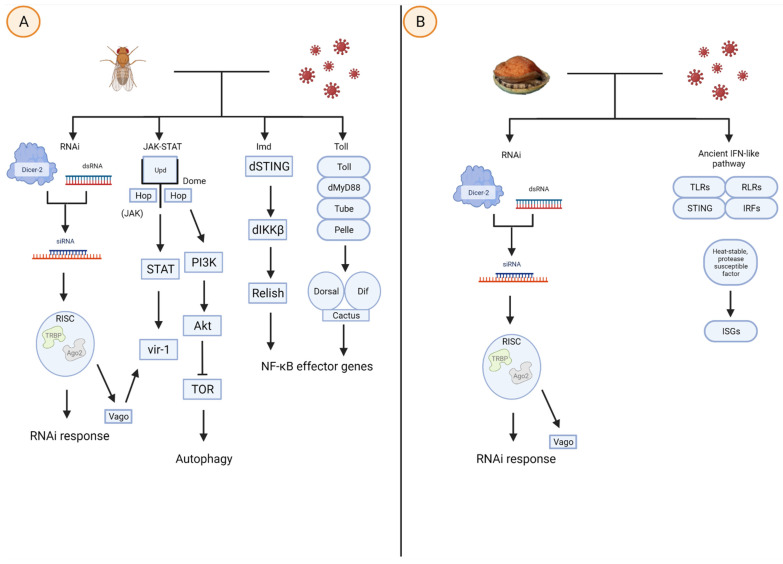
Viral infection in (**A**) *D. melanogaster* activates the RNAi, JAK-STAT, immune deficiency (Imd), and Toll pathways, whereas (**B**) molluscs rely on the RNAi pathway and homologs of an ancient IFN-like pathway. The RNAi involves Dicer-2 cleaving viral dsRNA to form siRNAs, which interact with RISC to seek and bind to complementary viral mRNA. The JAK-STAT pathway is activated when Upd cytokines bind to Dome, triggering JAK, and, subsequently STAT and *vir-1*, the latter of which may also be upregulated by RISC. The JAK-STAT pathway also upregulates PI3K and Akt to mediate TOR, which controls the autophagic response. The NF-κB effector genes, which in turn regulate cytokine levels and other transcriptional responses, can be upregulated following either the Imd or Toll pathway, the latter of which involves the intracellular signaling cascade of proteins such as dMyD88, Tube, and Pelle. Modified figure derived from [36,37] and created with BioRender.com.

It is critical to understand the homologous invertebrate IFN pathway and the mode of action of transcriptional responses to viruses (Figure 1). Such an understanding, as well as that of other effector molecules such as AMPs and lectins, may facilitate the manipulation of mollusc proteomes to promote the expression of various proteins, and enhance viral protection.

## 3. Antiviral and Antimicrobial Peptides

Antimicrobial peptides (AMPs) are a ubiquitous class of secretable molecules involved in innate immunity via direct interaction with pathogens. AMP research has sought to describe the highly conserved cysteine rich C-domains of peptides, which determine molecular function; however, investigations into such molecular functions have generally been limited to antibacterial and antifungal defence in both vertebrates and invertebrates, with little research focusing on mollusc antiviral AMPs. Mollusc AMPs can be broadly divided into the following five groups: defensins, big defensins, mytilins, myticins, mytimacins, and mytimycins, which are classified by their structure as well as the organisms they have been found in (Table 1). All groups possess antibacterial activity, though few have been tested for antiviral activity, and thus the mollusc antiviral AMP mode of action is poorly understood. However, proposed modes of action of antiviral AMPs include targeting viral entry, viral uncoating, and inhibition of viral replication and endosomal escape [38]. 

The majority of mollusc AMPs have been described in Gastropods and Bivalves, however, due to the conserved domains of the peptides, it has been hypothesised that structurally and functionally similar AMPs exist in other classes such as Polyplacophors, Cephalopods, and Scaphopods [49]. Further research is required to investigate the presence of homologous AMPs between mollusc classes, as well as between vertebrates and invertebrates, and to describe novel AMPs. 

### 3.1. Defensins and Big Defensins 

Defensins are the most characterised group of AMPs in both vertebrates and invertebrates, though no pure fractions of mollusc defensins have successfully been tested for antiviral activity. MGD-1, first isolated in *M. galloprovincialis*, is approximately 39 aa long and exhibits a common motif in defensins, termed an alpha-beta loop, consisting of an α-helix and two β-sheets [50,51]. Additionally, MGD-1 inhibits the growth of many Gram-positive bacteria by binding to lipid II, a precursor to peptidoglycan [50,52]. Though not tested for antiviral defence in molluscs, a 10 amino acid long fragment of MGD-1 has been demonstrated to inhibit mortality in the Chelicerate *Palaemon serratus* against White Spot Syndrome Virus by directly binding to and disrupting viral envelopes [44,53]. In these studies, a 10 amino acid long fragment of MGD-1 constrained by two disulphide bonds in a stable beta hairpin structure was shown to be the critical domain required for viral inhibition [44]. Moreover, MGD-1 breaks the conformity of other defensins such as the antibacterial MGD-2, possessing two additional C-domains, which is suggestive of a greater spectrum of molecular functions and renders the structure similar to Mytilus genus AMPs [44,51].

**Figure 2:**
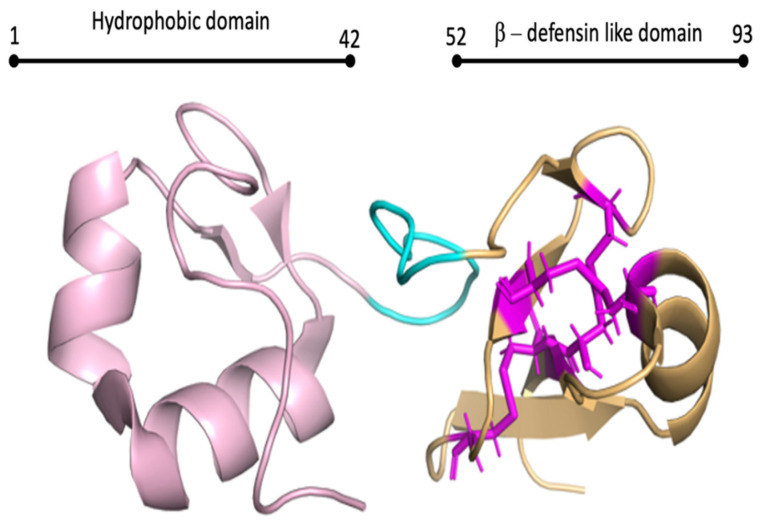
NMR structure of *Cg*-BigDef1 structure (6QBL). The N-terminal hydrophobic domain is represented in light pink (residues 1–42), the linker in cyan and β-defensin-like domain is light orange (residues 52–93). The critical cysteines that form disulfides are represented in mangta.

Big defensins are, as the word suggests, structurally larger (70–180 aa) than defensins, and possess both C-domains and an N-terminal [6,54] (Figure 2). Big defensins are believed to have given rise to vertebrate β-defensins, which lost the N-terminal during evolution from basal chordates to vertebrates [54]. The additional N-terminal of big defensins often provides additional protein functions, including antimicrobial activity [54]. To date, the mollusc big defensins have all only been tested for antibacterial and antifungal activity, including the three described in *C. gigas*; *Cg*-BigDef1, *Cg*-BigDef2, and *Cg*-BigDef3 [52,55]. Specifically, these three big defensins are active against both Gram-negative and Gram-positive species, and also exhibit bactericidal activity against *Staphylococcous aureus* multiresistant to antibiotics and *Vibrio* spp., by disrupting bacterial membranes through hydrophobic interactions [41,54].

Very few investigations have described big defensin diversity or gene organization, thus it is possible that there are yet undiscovered mollusc defensins that contain antimicrobial roles, including antiviral activity, which may be harnessed to manufacture both invertebrate and human infection treatments. 

### 3.2. Mytilus AMPs

Mytilus AMPs are those peptides isolated solely in members of the mollusc genus Mytilus [56]. These AMPs include the classes myticins, mytilins, mytimycins, and mytimacins [56]. All three identified myticins have expressed antibacterial and antifungal activity, but only myticin-C possesses antiviral activity [56] (Table 1). Myticin-C has gained the most attention for a single AMP, for its various roles in innate immunity. First isolated in the Blue Mussel (*M. galloprovincialis*), cysteine-rich AMP prevents the viral replication of OsHV-1 in molluscs and, interestingly, HSV-2 in humans [56]. Myticin-C was also shown to play a role in danger signaling, and is upregulated in response to tissue damage in *M. galloprovinicalis* [57]. Interestingly, myticin-C expresses a high nucleotide sequence variability across more than 100 mussels, perhaps suggesting that it is derived from a more ancient and common nucleotide sequence, and due to its highly conserved and ubiquitous nature, may play additional roles in innate immunity [58]. 

The five mytilin AMPs protect against bacterial and fungal infections, however, their antiviral roles are currently unknown [59]. Of the AMP class, mytilin-B is the most researched, due to its potent defensive roles against Gram-positive bacteria in *Mytilus edulis* [60]. It has been determined that mytilin-B contains an α-helix linked by four disulfide bridges to two β-sheets [61]. Though mytilin-B has not been tested for antiviral activity in molluscs, a 13 amino-acid-long fragment of one disulfide bridge and an acid loop structure has been tested against White Spot Syndrome Virus in the prawn, *P. serratus* [53]. The study found that the fragment, designed to mimic a common sequence found in defensins, offered minimal protection and animals died four days post challenge [53]. In comparison, whole mytilin-B provided an approximate 70% survival rate for 13 days post challenge, suggesting the mytilin-B possesses a separate structural attribute to other defensins that provides antimicrobial defence [53]. The structurally similar mytilin-A is also shown to inhibit herpes simplex virus-1 (HSV-1) replication during in vitro experiments, due to a dense cysteine-rich structure that competes for binding sites, and thus prevents viral attachment [60]. Therefore, it is possible mytilin-B and mytilin-A possess unknown antiviral roles in the Mytilus genus, the description of which may yield a broad spectrum of antimicrobial defence for all molluscs. 

Mytimycins and mytimacins are the least studied Mytilus genus AMPs and display antibacterial and antifungal activity, though they have not been tested for antiviral activity [6,62]. Mytimycins and mytimacins are generally larger than other Mytilus genus AMPs and defensins, at approximately 80–100 aa [6,61]. They have been shown to possess ten C-domains, which is more than any other Mytilus AMP [63], and as C-domains are related to peptide function, it is likely that the structurally similar mytimycins and mytimacins possess currently undescribed antiviral activity, which should be the focus of future studies [6]. 

### 3.3. Hemocyanins 

Hemocyanins (Hcs) constitute 50–90% of hemolymph and are copper-containing glycoproteins that transport oxygen, possess antimicrobial activity, and have C-domains that encode for peptides with AMP-like activity [64,65]. The antimicrobial activity of Hcs and equivalent glycoproteins, such as cavortin in *C*. *gigas*, include antiviral defence, as demonstrated by in vitro assays involving HSV-1 [66,67]. Purified abalone Hc (from *Haliotis rubra*), has been shown to bind to specific viral surface glycoproteins of HSV-1, inhibiting viral attachment and entry into Vero cells in vitro, however, it displayed no antiviral activity when added to the culture post HSV-1 infection [66]. 

The C-domain of Hcs encode for many Hc derived peptides (Hcdps), such as the highly conserved haliotisin, which is present in many of molluscs [68]. The C-domain of other proteins have been shown to encode for peptides with AMP-like activity, such as histone H2A in the abalone *Haliotis discus discus*, which encodes for the peptide abhisin [69]. Such domain-encoded peptides with AMP-like activity have only been tested for antibacterial and antifungal activity, with no investigations into antiviral properties. As these domain-encoded peptides are still relatively novel, investigations into their function remain very limited, and future research will hopefully include antiviral activity screens.

AMPs represent hopeful prospects for future mollusc innate-immunity studies, due to their highly conserved regions that aid in describing previously undiscovered peptides, as well as their role in antimicrobial defence. Moreover, AMP applications reach beyond mollusc innate immunity, and include the possibility of being repurposed to produce higher order species of antimicrobial compounds. Thus, investigations into mollusc AMPs are vital and should include a broader description of the functions and structures of currently undescribed peptides, and should also incorporate structural knowledge of AMPs to discover novel molecules. Such research may deepen our understanding of the mollusc innate immune system and the role of protein-derived effector molecules during viral challenge, thus supporting increased survival in farmed mollusc species such as abalone. Research may also lead to the engineering of antimicrobial treatments for a broad spectrum of both lower and higher order organisms. 

## 4. Antiviral Lectins

Previously known as agglutinins, lectins are secretable proteins involved in innate immunity by acting as additional PRRs to detect pathogens [70]. Molluscan lectin studies have mostly described antibacterial defence roles, with no investigations conducted into antiviral activity. Little research exists on the mode of action of mollusc lectins, although it has been hypothesised that these effector molecules function similarly to vertebrate lectins [71]. Vertebrate antiviral lectins, such as the human intracellular adhesion molecule 3 (ICAM3), reversibly bind to carbohydrate glyconjugates, such as glycoproteins, leading to pathogen degradation [72,73]. It has also been suggested that invertebrate lectins opsonize pathogens, thus allowing hemocyte phagocytes to degrade the target [71]. Mollusc lectins consist of seven groups, with Ca^2+^ dependent lectins (C-type lectins (CTLs)) and galectins being the most widely documented [74,75]. 

### 4.1. C-Type Lectins 

CTLs, similarly to other lectins, contain highly conserved regions known as carbohydrate-recognition domains (CRDs), which dictate signaling functions and have been used to isolate previously undescribed lectins [74]. CRDs exhibit a double loop structure, with the second loop containing four Ca^2+^ binding sites [76]. Carbohydrate binding occurs at ‘Site 2’, the affinity of which is determined by two conserved motifs [77]. There are three possible vertebrate motifs, which are as follows: EPN (Glu-Pro-Asn) or QPD (Gln-Pro-Asp) for the first, and WND (Trp-Asn-Asp) for the second [78]. Mollusc motifs are more diverse with eight currently described; EPN, QPD, EPD (Glu-Pro-Asp), QPG (Gln-Pro-Gly), QPS (Gln-Pro-Ser), YPG (Tyr-Pro-Gly), ENC (Glu-Asn-Cys), and YPT (Try-ProThr) [78] (Table 2). These motifs have displayed antibacterial activity, having been shown to bind to PAMPs such as peptidoglycan and lipopolysaccharides, although most individual functions remain elusive. 

Mollusc CTLs are diverse compared to their vertebrate counterpart; *C. gigas* possesses 266 CTLs, whereas humans contain approximately 100 [80,95]. This greater diversity of mollusc CTLs suggests that the already described or undiscovered CTLs may possess antiviral properties. Other invertebrates with CTLs expressing antibacterial activity also exhibit antiviral activity, such as the MnCTL described in Oriental River Prawn (*Macrobrachium nipponese*) [96]. MnCTL was able to clear the bacterial infections of *S. aureus* and *Vibrio parahaemolyticus* in *M. nipponese* individuals, the efficiency of which was enhanced by the addition of the recombinant protein MnCRD, suggesting that lectins function as part of a larger network [96]. In shrimp infected with White Spot Syndrome Virus, MnCTL mRNA was highly upregulated in the gills, potentially implicating its role in antiviral defence, though the mechanism remains unknown [96]. Therefore, due to the various immune functions of invertebrate CTLs and complexity and diversity of mollusc CTLs, it is likely that these effector molecules possess some form of antiviral defence. 

### 4.2. Galectins 

Galectins were initially described as proteins involved in early developmental processes, however, recent investigations suggest that lectins partake in several immunological functions, including acting as additional PRRs and inducing apoptosis [75,97]. Similar to CTLs, vertebrate galectins show both antibacterial and antiviral properties [98]. Specifically, galectin-1 has been shown to directly bind to surface glycoproteins and inhibit viral attachment of influenza A/WSN/33, thus preventing hemagglutination activity [98]. Galectin-3 can bind to lactosyl or lipid A moieties of bacterial lipopolysaccharide (LPS) to both disrupt pathogen-host interacts and induce an inflammatory response [99].

Galectins are divided into the following three subgroups in vertebrates: (1) prototype, that contain one CRD and possess the ability to form homodimers; (2) tandem-repeat galectins, which have two closely linked CRDs; and (3) chimera-type galectins that show one CRD with a non-lectin domain [75,100]. Mollusc galectins do not conform to vertebrate domain organization, and instead are classified by the number of CRDs they possess, pertaining to 1-CRD, 2-CRD, and 4-CRD galectins [75]. These vary in size, at approximately 140, 280–300, and 560aa long, respectively, compared to the consistent 120–130aa galectins of vertebrates, representing the complexity and variance of mollusc galectins [80,101,102]. 

Galectins in molluscs have been shown to also act in a PRR-like manner, however their direct anti-pathogen roles have been mainly tested in response to bacterial and parasitic challenge, while very few investigations into the antiviral properties of mollusc galectins have been conducted [80,103]. Although a recent transcriptome analysis of oysters has shown that galectins are only upregulated following bacterial challenge, but absent during a viral challenge [104], a recent study showed that a quadruple galectin (AbGalec) from *H. discus discus* was upregulated by polyinosinic: polycytidylic acid (Poly I:C) and viral hemorrhagic septicemia virus (VHSV) injections [105]. In addition AbGalec was also able to neutralize VHSV infection within an in vitro culture system via an unknown mechanism [105]. It is clear that research into the anti-viral properties of this diverse family of novel galectins in molluscs is very limited, and moving forward, research should seek to investigate the potential antiviral properties of these novel proteins.

### 4.3. Other Lectins 

Other lectin groups include fucose-binding proteins lectins (F-type lectins (FTLs)), ficolin-like proteins, and chitinase-like lectins [106]. Various studies have shown these groups to be upregulated during bacterial challenge in mollusc species, such as *Pincata martensii* and *Crassostrea hongkonggensis* in response to *Vibrio alginolyticus* and *Escherichia coli*, respectively, as well as during viral challenge in *Haliotis iris* when exposed to HaHV-1 [106,107]. However, the exact mechanism of action these lectins play in antiviral defence remains largely unknown. Future studies should aim to further determine the structure and function of currently described lectins. Further investigations may also seek to discover novel lectins or those homologous to vertebrates, possibly leading to a clearer depiction of the mollusc innate system. Such investigations may allow for the manipulation of the mollusc proteome to improve longevity against viral infection. Moreover, additional roles of currently described PRRs may be identified, resulting in heightened immunity against a broader spectrum of antimicrobial infections. 

## 5. Future Directions and Applications of Mollusc Proteome-Encoded Effector Molecules 

Many assumptions regarding the mollusc antiviral defence and proteome-encoded effector molecules have been derived from model organisms, however, recent literature exhibits many divergences between the mollusc and model organism immune systems [107]. Although general hemolymph properties may be examined in molluscs, molecular studies have proven challenging, and information regarding their proteome-encoded effector molecules remains unknown. Investigations have been inhibited due to the difficulty of developing cell lines in a laboratory setting and restricted access to draft invertebrate genomes [101]. 

However, there is increasing importance placed on marine mollusc production as a sustainable and environmental food product. This will in turn drive interest in developing tools to investigate these animals [102]. Among the mollusc proteome-encoded effector molecules, proteins, AMPs, and lectins are poorly understood compared to their vertebrate counterparts, though they represent a valuable focus for future study. As such, the advent of more mollusc genomes being sequenced will allow bioinformatic approaches to identify various effector molecules with anti-viral activity such as antimicrobial peptides [108]. 

Research is required to detail the specificities of mollusc innate immunity, which may enable the manipulation of mollusc proteomes to provide a strengthened antiviral response to significant viral pathogens, such as herpesviruses. Many different viruses cause major health problems in livestock and humans thus anti-viral effectors such as lectins and AMPs from molluscs could be developed as novel treatments that interfere with viral entry and replication.

Further research into understanding the mechanism of viral-mollusc interactions will enhance our ability to prevent significant economic loss in the industry and further develop the use of molluscs as a model system for human health [109].

## Figures and Tables

**Table 1:** Summary comparison of AMPs and lectins. Columns describe effector molecule, protein class, example species, protein physical characteristics, mollusc proteins with antiviral activity, and references. * indicate molecules with antiviral activity. Table derived from [6,35,39,40,41,42,43,44,45,46,47,48].

Soluble Mediator	Protein Class	Example Species		General Protein Physical Characteristics	References
**Defensins**	AMPs	Molluscs	*M. galloprovincialis* (MGD-1 and MGD-2)	Size: 18–60aa C-Domains: 6, hydrophobic, 3–4 disulfide bridges, α-helix linked to two stranded β-sheets.	[39,40,41,42]
		Invertebrates	*D. melanogaster* (Drosomycin)	Size: 20–70aa C-Domains: 6, hydrophobic, 3–4 disulfide bridges, α-helix linked to two stranded β-sheets.	[39]
		Vertebrates	Humans (HBD-2 and HBD-3)	Size: 70–120aa C-Domains: 6, hydrophobic, 3 disulfide bridges, α-helix linked to three stranded β-sheets.	[39,40,41]
**Big Defensins**	AMPs	Molluscs	*C. gigas* (BigDef1-3)	Size: 70–180aa C-Domains: 6, hydrophobic, 3 disulfide bridges, α-helix linked to two stranded β-sheets. * Additional hydrophobic N-domain.	[6,39,40,41]
		Invertebrates	*Tachypleus* *tridentatus* (Big Defensin)	Size: 70–120aa C-Domains: 6, 3 disulfide bridges, α-helix linked to two stranded β-sheets. * Additional hydrophobic N-domain.	[6,39,41]
		Vertebrates	N/A	N/A	
**Myticins**	AMPs	Molluscs	Only Mytilus genus; *M. galloprovincialis, M. edublis* (myticin-C)	Size: 30–100aa C-Domains: 8, hydrophilic, 4 disulfide bridges α-helix linked to two stranded β-sheets.	[6,39,43]
**Mytilins**	AMPs	Molluscs	Only Mytilus genus; *M. galloprovincialis, M. edublis* (mytilin-B)	Size: 30–100aa C-Domains: 8, hydrophilic, 4 disulfide bridges α-helix linked to two stranded β-sheets.	[6,42,43,44]
**Hemocyanin**	AMPs	Molluscs	*Haliotis discus discus*	Size: 350–450aa C-domains: 7–8 C, hydrophobic, 3 disulfide bridges, α-helix linked to two stranded β-sheets. * Additional hydrophobic N-domain.	[35,45,46,47,48]
		Invertebrates	Mostly arthropods; *Limulus polyphemus*	Size: 350–450aa C-domains: 7–8 C, hydrophobic, 3 disulfide bridges, α-helix linked to two stranded β-sheets. * Additional hydrophobic N-domain.	[45]
		Vertebrates	N/A	N/A	

**Table 2:** Mollusc motifs determining the affinity of carbohydrates binding to ‘Site 2’ of CRDs. The first column indicates the seven described motifs. The second column displays CTLs that express the various motifs, and the third column lists mollusc species containing the CTLs and motifs. Table derived from [77,78,79,80,81,82,83,84,85,86,87,88,89,90,91,92,93,94].

Motif	Example CTLs	Mollusc Species	Reference
EPN	Codakine Cflecs AiCTLs	*Codakia orbicularis* *Chlamys farreri* *Argopecten irradians*	[78,79,80,81,82,83,84,85]
QPD	MCL-3 AiCTLs	*Ruditapes* *philippinarum* *Argopecten irradians*	[78,86,87,88]
EPD	Clfecs AiCTLs	*Chlamys farreri* *Argopecten irradians*	[78,83,89,90,91]
QPG	CLHd MeML CvML	*Haliotis discus discus* *Mytilus edulis* *Crassostrea virginica*	[78,92,93]
QPS	CLHd MeML CvML	*Haliotis discus discus* *Mytilus edulis* *Crassostrea virginica*	[78,92,93]
YPG	CLHd MeML CvML	*Haliotis discus discus* *Mytilus edulis* *Crassostrea virginica*	[77,78,92,93]
ENC	MeML	*Mytilus edulis*	[77,78]
YPT	Cflecs AiCTLs	*Chlamys farreri* *Argopecten irradians*	[77,83,94]
